# Retraction Note: Synthesis and characterization of trimeric phosphazene based ionic liquids with tetrafluoroborate anions and their thermal investigations

**DOI:** 10.1038/s41598-021-85542-6

**Published:** 2021-03-09

**Authors:** Ahmet Karadağ, Hüseyin Akbaş, Ali Destegül, Çiğdem Çakırlar, Yusuf Yerli, Zeynel Kılıç, Fatih Sen

**Affiliations:** 1 Department of Chemistry, Faculty of Arts and Sciences, Yozgat Bozok University, 66200 Yozgat, Turkey; 2 Department of Chemistry, Faculty of Arts and Sciences, Gaziosmanpaşa University, Tokat, Turkey; 3 Department of Physics, Gebze Technical University, Kocaeli, Turkey; 4 Department of Physics, Yıldız Technical University, Istanbul, Turkey; 5 Department of Chemistry, Ankara University, Ankara, Turkey; 6 Department of Biochemistry, University of Dumlupınar, Kütahya, Turkey

Retraction of: *Scientific Reports* https://doi.org/10.1038/s41598-020-68709-5, published online 16 July 2020

The Editors have retracted this article.

After publication concerns were raised that some of the NMR spectra contain irregularities. Specifically:

- There are repetitions in the noise signal, which cannot be accounted for by the way the experiments were performed, in the ^31^P NMR spectrum for PzIL1a in Figure 1, the ^31^P NMR spectrum for PzIL8a in Figure S1 and the ^31^P NMR spectrum for PzIL6a in Figure S2. The Authors were not able to provide the original data for these spectra;
- There are repetitions in the noise signal, which cannot be accounted for by the way the experiments were performed, in the ^31^P NMR spectrum for PzIL5a in Figure S1. The Authors provided original data for this spectrum, which showed that original data does not match Figure S1;
- The Authors were able to provide original data for some of the spectra for samples PzIL2a, PzIL4a and PzIL5a. However, analysis of these data showed that the original spectra contained peaks that are not present in Figures S4 and S12.

The Editors therefore no longer have confidence in the integrity of the data presented.

Ahmet Karadağ, Hüseyin Akbaş, Ali Destegül, Çiğdem Çakırlar, and Yusuf Yerli disagree with the retraction. Zeynel Kılıç and Fatih Sen did not respond to the correspondence about this retraction.

